# Fat Embolism Syndrome Mimicking a COVID-19 Infection

**DOI:** 10.1155/2021/5519812

**Published:** 2021-05-06

**Authors:** Alexandru Leonard Alexa, Adela Hilda Onutu

**Affiliations:** ^1^1st Department of Anaesthesia and Intensive Care, Iuliu Haţieganu University of Medicine and Pharmacy, Cluj-Napoca, Romania; ^2^Department of Anaesthesia and Intensive Care, The Regional Institute of Gastroenterology and Hepatology “Prof. Dr. Octavian Fodor”, Cluj-Napoca, Romania; ^3^Orthopedic Anaesthesia and Intensive Care Compartment, Emergency County Hospital Cluj-Napoca, Romania

## Abstract

Fat embolism syndrome (FES) is a multiple organ disorder that can appear after pelvic and long bone fractures. The most common clinical finding is hypoxia, accompanied by diffuse petechiae, alveolar infiltrates, altered mental status, fever, polypnea, and tachycardia. We present a mild FES case on a 32-year-old man with no medical history admitted for an orthopedic procedure, following both tibia and fibulae fractures. Thirty hours postoperatively, he developed respiratory failure with altered mental status and needed admission in the intensive care unit. The chest radiography and later chest tomography raised the suspicion of a COVID-19 disease, even if our first suspicion was FES. After being carefully investigated in a dedicated COVID-19 ward and three negative RT-PCR SARS-CoV-2 tests, he returned to continue supportive treatment in the orthopedic intensive care ward. His evolution was favorable with discharge at ten days, without sequelae. In the context of the SARS CoV-2 pandemic, differential diagnosis has become an increasingly challenging process. Added to the variety of preexisting respiratory diseases and disorders, the COVID-19 infection, with its symptomatology so similar to multiple other pulmonary diseases, must not cloud our clinical judgement.

## 1. Introduction

Fat embolism syndrome (FES) is a multiorgan potentially lethal disorder commonly seen in polytrauma patients, especially in those with pelvic and long bone multiple fractures [1].

In the orthopedic traumatological wards, we encounter patients admitted for surgical intramedullary fixation or other procedures that imply canal reaming and handling pedicle screw.

The condition was first described in 1861 by Zenker as a syndrome with neurological, cutaneous, respiratory symptoms in long bone fracture patients. Later, in 1878, Bergmann presented the first clinical case of fat embolism syndrome in a patient who was suffering from a distal femoral fracture [2].

Fat embolism syndrome can present itself clinically in a great variety of severity and symptoms. They tend to appear 12 to 72 hours after the causal event, not always with the typical triad of respiratory distress, petechiae, and mental status change [3].

## 2. Case Report

We presented the case of a 32-year-old ASA I, average weighted man who admitted in our orthopedic ward with an open type II fracture in the mid-third of both the tibia and the fibula. After giving informed consent and preparation, he went for intramedullary nailing. The surgery was uneventful, with normal respiratory and hemodynamic parameters, under spinal anesthesia.

**Figure 1:**
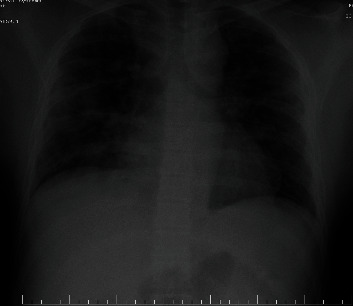
Patient's chest X-ray.

Thirty hours after surgery, on the surgical ward, the patient developed hypoxia (Sat O2—64%), chest pain, and productive cough accompanied by anxiety and was admitted to the Intensive Care Unit (ICU). Before the admission, a chest X-ray was taken (Figure 1) and showed multiple infiltrates, mainly on the right lung. After being monitored and oxygen therapy by face mask at a FiO2 40% started, we took a blood gas analysis sample. The latter showed respiratory alkalosis and a PaO_2_ of 79.9 mmHg thus a PaO_2_/FiO_2_ ratio of almost 200 mmHg. At admission, the patient presented tachycardia (120 beats per minute) and stable blood pressure (120/63 mmHg) and did not require any vasoactive or inotropic support. We administered 8 mg dexamethasone and 5000 UI sodic heparin as an intravenous bolus. After that, he received heparin subcutaneously and dexamethasone, in the mentioned doses, intravenously every 8 hours. CPAP was also started at 4-hour intervals in 10 minutes reprises, and after 6 hours, the patient had improved his respiratory status, and the PaO2 and SaO2 returned to normal values. The biological parameters showed mild anemia and high values for IL-6 and PCR.

**Figure 2:**
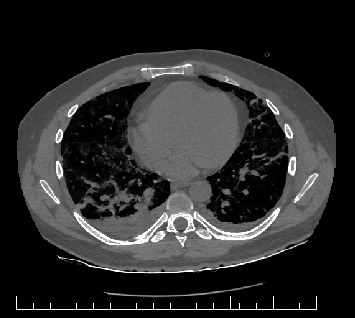
Patient's CT scan.

The following day, the patient had a thoracic computer tomography scan (Figure 2), and the radiologist doctor raised the suspicion of SARS-CoV-2 infection. The CT scan showed multiple zones of ground-glass opacities in both lungs, some of them with alveolar fillings, the largest one right posterior basal. Apart from a small bilateral pleural effusion, there were no signs of pulmonary embolism.

Next, we took a real-time PCR sample and sent it for examination, and consecutively, the patient was transferred to a dedicated COVID-19 ICU. After three negative RT-PCR SARS-CoV-2 tests and a biological assessment in the infectious disease ward, the patient returned to our ICU to continue the supportive treatment.

Eventually, we confirmed the fat embolism diagnosis. The patient had a favorable evolution and was discharged after ten days of hospitalization. Prior to his discharge, he gave his written informed consent to publish his medical case.

## 3. Discussion

In the COVID-19 pandemic, every respiratory syndrome must raise the suspicion of infection with the SARS-CoV-2 virus. In the orthopedic surgery ward, respiratory failure usually means pulmonary embolism, or bronchopneumonia in older patients.

The explanation of resembling between the two respiratory manifestations could be the one advanced by Cinti and collaborators that in the severe forms of COVID-19 developed by obese patients, fat emboli could trigger the lungs' inflammatory mechanisms [4].

When considering a respiratory failure diagnosis, we should also take into account a medical, nonsurgical, nontraumatic cause of fat pulmonary embolism. Having the same clinical symptoms, with a highlight over the neurological features and no traumatic involvement, fat embolism syndrome could be expressed as a cerebral fat embolism, the latter one being able to worsen the prognostic of the patients. Nevertheless, in a cerebral fat embolism, neurological manifestation may mask respiratory symptoms or they may even be absent, making diagnosis challenging [5–8].

According to Schonfeld's Fat Embolism Score Index, our patient had 7 points (from 16 maximum) and raised the suspicion of FES. A score of 5 points or more in the first days of hospitalization indicates a FES (Table 1) [9].

A recent study reported a case admitted with the suspicion of COVID-19 in a patient with tibial fracture. Later after collecting all data, despite the radiologic similarities, the final diagnosis was FES [10].

Oppositely, in our case, the first symptoms that lead us to believe that we are dealing with a FES were hypoxia and tachycardia, in a patient with recent tibial surgery. Complimentary, a high-resolution chest tomography revealed alveolar infiltrates, and the radiologist's opinion was different. They firmly believed it to be viral, and COVID-19 infection was their suspicion. It should be mentioned that at that particular time frame, patients were not tested for COVID-19 upon admission in the hospital, only those who had symptoms of infection or had a contact history with a positive COVID-19 patient. It could have been obtained a confirmation of FES by neutral lipid concentration from bronchoalveolar lavage. It would help us establish a precise and fast diagnosis of FES, but it was not possible as the patient was not intubated, and even so, COVID-19 could not have been excluded.

Radiological scoring systems were developed in order to help clinicians in differentiating between a COVID-19 infection and a non-COVID-19 pneumonia, but the analysis encountered justified limitations and further multicenter trials will be able to identify a high specificity scoring system [11].

In FES management, certain aspects require careful attention. These include prevention, when possible, fracture management, supportive care, and shock treatment. Human albumin remains one of the most used volume resuscitation choices, and there is evidence of its effects on binding fatty acids to decrease the level of lung injury [3, 12].

Orthopedic surgery techniques are in permanent development to prevent FES. In the desired attempt to reduce the intramedullary pressure during surgery, new techniques replaced traditional methods, such as slow insertion of hollow nails, narrower reamers, distal venting, and reamer irrigation-aspiration devices [13–15].

In our case, we decided to administer dexamethasone to reduce the inflammatory response. Studies showed a significantly reduced risk of hypoxia while administering corticosteroids. Low-dose regimens registered more benefits over the high-dose regimens in patients developing ARDS. Still, no significant differences were found in terms of mortality or over the rates of infection [12, 16].

Hypoxia is improved by oxygen therapy in FES patients. Numerous devices can deliver oxygen to a precise FiO_2_. If PaO_2_ is not improving with standard gas delivery systems, then high flow nasal cannulas or continuous positive pressure ventilation (CPAP) could improve the patient's outcome without a significant increase in FiO_2_. However, some severe cases of FES may require mechanical ventilation. When mechanical ventilation is initiated, one must consider ventilator-induced lung injury and the increase in right ventricular pressure, which will decrease the cardiac output [2].

Clinical similarities have been added when discussing fat embolism and COVID-19 infection through a radiologist or a pathologist perspective, even more challenging to differentiate from the two of them when linked to obesity or overweight patients [2, 4].

Considering that we always find ourselves in need of a precise diagnosis and next phase care for our patients, the FES should be a diagnosis of exclusion, because so far, clinical and sometimes radiological similarities between them can be misleading.

## 4. Conclusion

We should suspect fat embolism syndrome in all patients who develop respiratory failure after long bone fracture surgery. This case report highlights the difficulty of respiratory pathology diagnosis in the new COVID-19 pandemic time. While this virus does not seem to back down, we will have to adapt and accept various respiratory diseases, which add a high mortality risk.

## Figures and Tables

**Table 1:** Schonfeld's Fat Embolism Score Index [5].

Symptoms and signs	Points
Diffuse petechiae	5
Alveolar infiltrates	4
Hypoxemia (under 70 mmHg)	3
Confusion	1
Fever (above 38 degrees Celsius)	1
Heart rate (over 120 bpm)	1
Respiratory rate (over 30 RR)	1
Fat embolism score: 5 or more points	

## Data Availability

The data supporting this case report can be released with acceptance of the authors.
